# National bitterness, powerlessness and greatness: Examining constructions of affect as part of argumentation in populist EU discourse in Finland

**DOI:** 10.1111/bjso.70055

**Published:** 2026-02-12

**Authors:** Helenor Tormis, Inari Sakki, Katarina Pettersson

**Affiliations:** ^1^ Department of Social Sciences University of Helsinki Helsinki Finland

**Keywords:** affect, critical discursive psychology, EU, Europe, intergroup relations, national identity, rhetorical psychology

## Abstract

Social psychological research exploring the rhetoric of Eurosceptic, right‐wing populist actors and laypeople's argumentation in the polarizing context of Brexit has indicated the emotion‐laden nature of EU‐related issues. However, few studies have explicitly united affective and discursive psychological analyses of these topics. To fill this gap, the present study expands the discursive psychological approach to consider the interplay between affect and discourse in populist argumentation around the EU. The study utilizes qualitative interviews with 31 voters in Finland who supported or did not oppose an EU‐critical statement from a radical right populist party's programme. We identified three key affective‐discursive practices: (1) bitterness towards the undeserving ‘other’ justifying opposition to the EU; (2) national uncertainty mitigating criticism of the EU; and (3) national glory devaluing the EU. This study contributes to social psychological research on affect by demonstrating how rhetorical and discursive resources—particularly national referents, temporality, history—are employed in constructing affect as intertwined with identities and intergroup relations around EU issues. Instead of approaching it as implicit or marginal, this framework enables a thorough consideration of affect in argumentation around political issues, providing an in‐depth understanding of its moral, relational and contextual nuances.

## INTRODUCTION

Over the past two decades, the European Union (EU) has faced numerous political challenges—including the financial crisis, the refugee ‘crisis’, Brexit, the outbreak of COVID‐19 and the war in Ukraine. Radical right populist parties across Europe have seized on these events to mobilize Eurosceptic, critical views, gaining further electoral support. An extensive study on voter turnout in European Parliament (EP) elections has shown that after decades of decline, the turnout has surged in recent years due to the politicization of European integration and the rise of Eurosceptic political forces at the national level (Kostelka & Krejcova, 2026). Perhaps the most striking example of the consequences of such political voices comes from the British Isles, where the UK Independence Party (UKIP), under the leadership of Nigel Farage, mounted a fierce campaign against the EU that culminated in the 2016 referendum to leave the bloc.

The EU—a union of 27 nation‐states established in the aftermath of World War II to promote peace and economic cooperation among European nations (European Union, 2024)—makes a compelling context for social psychological study, because it highlights the negotiation of multiple (inter)national identities. In the British context, Henkel (2021) showed how the decades‐long false reporting of the EU policies by the British media created an Eurosceptic myth that naturalized the claim of antagonistic British‐European relations wherein the witty Briton must stand up against the European threat. This is important because the media and the rise of Eurosceptic political forces are powerful in shaping the image of a nation, its people and their relations with other member states in this multinational union. Previous social psychological research on the EU, particularly on Brexit, has shown how citizens not only negotiate these multiple identities and intergroup relations, but also balance between the positive and negative aspects of the EU membership (Andreouli & Nicholson, 2018; Sullivan, 2021).

The negotiation of (inter)national identities and the contested nature of political issues, such as the EU, highlight how emotions are deeply implicated in such identities (Sakki et al., 2024; Smeekes & Verkuyten, 2015; Smith, 1993), implying the essential role of emotions within EU issues. Although the EU is not the only emotion‐laden political issue, previous research examining the discourse of parties and voters critical of the EU has demonstrated that it is often portrayed as a threat to national sovereignty, culture and economic stability (Mudde, 2007; Sullivan, 2021; Wodak & Boukala, 2015), with immigration as a key point of argumentation (Andreouli et al., 2019, 2020; Andreouli & Nicholson, 2018; Sullivan, 2021). Such examples suggest that various societal developments – such as European integration, that is, the economic, social and political processes of mutual accommodation and inclusion among European states and peoples (Manners, 2014, p. 264)—are often portrayed as undermining the continuation of national identity and sovereignty (Smeekes & Verkuyten, 2015). National concerns remain important elements in citizens' evaluation of the EU (Taggart, 1998), thus highlighting and amplifying its emotion‐laden and contested nature (Manners, 2018; Sullivan, 2021).

Moreover, previous qualitative social psychological studies in the European context have focused on political debates, media discourse (Demasi, 2019; Demasi & Tileagă, 2021; Goodman & Narang, 2019; Henkel, 2021; Hunt & Demasi, 2025; Wodak & Boukala, 2015) or on voters' argumentation in the highly polarized context of Brexit (Andreouli et al., 2019; Andreouli & Nicholson, 2018; Sullivan, 2021), overlooking an important research perspective: the everyday argumentation of EU‐critical populist supporters in a generally EU‐positive Nordic context. Considering the rise of EU‐sceptic voices across Europe and the consequences of such voices, as in the case of Brexit, there is a need for a more fine‐grained social psychological understanding of the grievances, feelings and reasoning around these issues in times of uncertainties and global threats. Therefore, this study examines affective argumentation of voters who support or do not oppose a populist EU‐sceptic message in Finland and pays particular attention to how emotions and affect, understood qualitatively as social, relational and situated concepts (Wetherell, 2012), are bound up with political talk on the EU and European issues. By studying the political thinking of lay people, we can illuminate the multitude of everyday affects around EU issues and highlight their social and political consequences.

## THEORETICAL FRAMEWORK

### The EU as an emotion‐laden issue in political thinking

Emotions play a crucial role in our political and social world. They are linked to various political phenomena, particularly right‐wing populism (Demertzis, 2006; Nguyen, 2019; Salmela & von Scheve, 2017, 2018). Research has characterized right‐wing populist politics as politics of fear (Wodak, 2015) and has identified diverse emotions, such as fear, anger (Nguyen et al., 2022; Rico et al., 2017), shame, insecurity, hatred towards perceived enemies (Salmela & von Scheve, 2017), *ressentiment*, bitterness (Capelos & Demertzis, 2022; Salmela & Capelos, 2021) and a sense of dominant group victimhood (Reicher & Ulusahin, 2020) as key factors in populist appeal. More specifically, prior research examining emotions in relation to attitudes towards the EU has found that fear increased British citizens' will to renegotiate their constitutional relationship with the EU, while anger increased their willingness to leave (Vasilopoulou & Wagner, 2017). Similarly, Garry (2014) examined EU attitudes in Ireland, revealing the different implications of fear and anger on voting choices in the Irish referendum. Beyond these common emotions, Lantos and Forgas (2021) identified collective narcissism—the belief in the greatness of one's ingroup and entitlement to privileged treatment (De Zavala et al., 2009)—as linked to negative attitudes towards the EU and support for Hungary's ruling anti‐EU party, Fidesz.

Capelos and Katsanidou (2018) criticize the use of limited discrete emotion categories, such as fear or anger, in understanding political decision‐making and advocate a more complex and nuanced approach to affectivity in understanding political orientations. Using data from the European Social Survey from 2004 and 2014, they demonstrated that, in 2004, Eurosceptic preferences were primarily influenced by general dissatisfaction with life, low social and institutional trust and a decline in voting, indicating disengagement. However, by 2014, Euroscepticism was driven by dissatisfaction and distrust, but also by feelings of unfair treatment, right‐wing positioning and traditional values, suggesting an increase in *resentful* sentiment. This approach highlights the need for moving beyond discrete emotion categories towards a more complex understanding of affectivity.

Another such recent development by Capelos et al. (2025) draws on notions from psychoanalysis and psychology and introduces the concept of ‘anti‐social triad of grievance politics’. This triad includes reactionism (a backwards‐facing political orientation), *ressentiment* (an embittered emotional mechanism) and collective narcissism (De Zavala et al., 2009), which are claimed to be psychologically interrelated and linked to populist attitudes and anti‐preferences, making a compelling theoretical background to understand affects as part of reactionary grievance politics.

Despite these novel approaches, previous research has predominantly relied on quantitative measures and more cognitive approaches that view emotions and affect as fixed emotion categories (Garry, 2014; Nguyen et al., 2022; Rico et al., 2017; Vasilopoulou & Wagner, 2017) or individual inner states (Capelos et al., 2025; Capelos & Demertzis, 2022), paying less attention to a more qualitative, contextual, social and nuanced constructions of affect and emotions in political talk on contested issues (for exceptions see Andreouli et al., 2019; Sakki et al., 2024; Sullivan, 2021; Tormis et al., 2024). Therefore, there is a need for approaches that examine affect explicitly as part of political argumentation embedded with (inter)national identities at the ‘grassroots level’.

### A critical discursive psychological approach to affect and emotions

To examine the contested and emotionally charged nature of EU issues qualitatively, this study draws on a critical discursive psychological framework (Edley, 2001; Wetherell, 1998) that employs Wetherell's (2012) conceptualization of affect and Billig's (1987, 1988) rhetorical psychology. Critical discursive psychology builds on the foundation of discursive psychology, which differs from more traditional social psychological approaches in placing language, or discourse, centre‐stage (Wetherell & Edley, 2014). This means that while mainstream psychology typically treats language as providing access to an individual's psychological states, such as attitudes and emotions, discursive psychology examines how people talk about, or in other words, construct concepts such as identities, attitudes and emotions (Antaki & Widdicombe, 1998; Edwards, 1999; Potter, 1998; Potter & Wetherell, 1987). According to discursive psychology, speakers invoke psychological states to construct versions of reality (Edwards & Potter, 1992). The *critical* discursive social psychology adopted in this study looks at the micro‐level constructions and negotiations of psychological states while *also* describing the macro‐level collective and social patterning of the normative context. Therefore, it is interested in the flexible construction of identities through language (Edley, 2001), situated within a broader ideological setting (Wetherell, 1998).

With respect to the EU, previous research has adopted a discursive psychological framework to study Brexit issues in parliamentary and political debates (Demasi, 2019; Hunt & Demasi, 2025) and in the media (Goodman & Narang, 2019), or by looking at the experiences of Brexit voters and non‐voters (Andreouli, 2018; Andreouli et al., 2020; Andreouli & Nicholson, 2018; Sullivan, 2021). In the media, Goodman and Narang (2019) examined online comments of the British newspaper *the Daily Mail* and showed how arguments about child refugees as financial burdens or not as children were used in Brexit debates to build a case that presented leaving the EU as the solution to the migration problem. In political debates, Demasi (2019) studied how politicians in the UK used fact‐based (counter)claims in multiparty interactions, demonstrating the strategic use of ‘fact’ and ‘truth’ when debating the EU (Demasi, 2019). To build upon and expand this work, a number of studies have called for a more explicit examination of the affective nature of the EU discourse (Andreouli et al., 2019; Sakki, 2010; Sakki et al., 2021; Sullivan, 2021).

Wetherell's (2012) affective‐discursive approach provides an appropriate framework for such an endeavour, which emphasizes the key role of affect in discourse. Consistent with affective practice theory (Wetherell, 2012), emotions and affect^1^ are examined within their sociopolitical contexts, linking affect to everyday discourse. From this perspective, emotions are not perceived as objectively *felt* clear emotion categories, such as anger or sadness. Instead, the concept of affective‐discursive practice is used to understand emotions and affect as part and parcel of the discursive meaning‐making, including social relations, the flow of ordinary life and the sociopolitical context (Wetherell, 2012). In line with critical discursive psychology (Wetherell, 1998), this approach draws on the micro‐level interactions in everyday social life *and* the macro‐level ideologies and power relations, differing from Edwards' (1999) work on emotion discourse as a more micro‐level phenomenon that focuses on how emotion *words* and *categories* can serve and be worked up as rhetorical resources in talk to accomplish interactional functions, such as explaining, building or undermining people's actions. Wetherell's (2012) approach has previously been applied to the analysis of social issues in interview settings (Haugestad & Carlquist, 2025; Sullivan, 2021; Tormis et al., 2024; Venäläinen & Calder‐Dawe, 2024), and it shows potential in examining emotion‐laden political discourses (Hokka & Nelimarkka, 2020; Sakki & Martikainen, 2021; Venäläinen, 2022). For example, Tormis et al. (2024) employed this approach to examine populist environmental discourse among right‐wing populist supporters in Finland. The findings demonstrated the constructions of annoyance and irritation towards the ‘pro‐environmental other’ and unfairness towards ordinary rural people, ‘us’, which functioned to belittle climate action and claim victimhood for one's ingroup, respectively. Therefore, emotions and affect intertwined with social relations have particular social implications in interaction.

In the EU context, Sullivan (2021) used Wetherell's (2012) approach in studying UKIP supporters and non‐voters and focused explicitly on whether, and how, they demonstrate resentment and ressentimenful affectivity in their talk. The study showed how emotions, such as anger, resentment and fear, were combined with multiple anti‐political preferences, including opposing voting, leaving the EU and immigration (Sullivan, 2021). Additionally, it demonstrated that shame‐repression and nostalgia for a relatively prosperous past worked as key emotions in UKIP voters' anti‐EU sentiments stemming from the UK politicians' disregard in relation to Europeans or the EU.

Other qualitative studies have highlighted the importance of affect and focused similarly on Brexit issue in the UK. For example, using a rhetorical psychological approach, Andreouli and Nicholson (2018) demonstrated how the distinction between pragmatism/reason and emotion was used to argue in favour of the EU in focus group discussions on the EU referendum. In another study, Andreouli et al. (2019) revealed that Remain voters' discourse often relied on rational and economic arguments based on cost/benefit calculations, while Leavers constituted affective citizens, imbued with emotion, including a sense of outrage and righteous anger towards the threatening ‘other’, including politicians, the EU and immigrants. This was expressed through a passionate tone, extreme expressions and a disregard for facts. Although Andreouli et al. (2019) argued that both sides of the Brexit debate exhibited two styles of argumentation—rational, consensus‐seeking and fact‐oriented vs. impassioned, general and conflict‐based, Manners (2018, p. 1228) cautioned against simplistically contrasting rationality with emotions, advocating for a more critical and nuanced social psychological understanding of EU issues. Therefore, what is missing in previous research is a consideration of more subtle everyday constructions of political affect interwoven in the nuanced work of social categories, beyond the rational vs. irrational Brexit context. While building on this discursive theorization of affect and emotions, the present study also aims to expand Wetherell's (2012) affective practice theory and use the concept of *affective argumentation* to pay closer attention to affect and emotions as intertwined with argumentation around contested sociopolitical issues, such as the EU. With a focus on argumentation, it draws on rhetorical psychology (Billig, 1987, 1999), which similarly views emotions—such as love and anger—as discursive practices embedded in language use and bound up in social, cultural and ideological relations. This perspective connects affect and emotions more closely to argumentation, positing that, as part of thinking, emotions can be understood in terms of dialogue between arguments and counterarguments (Billig, 1999).

The rhetorical psychological approach acknowledges that human thinking is often inconsistent, fragmented and even contradictory, reflecting *ideological dilemmas*, meaning the opposing ways of discussing a social issue or situation in common‐sense thinking (Billig et al., 1988). From this perspective, the conflicting ideological dilemmas in our everyday thinking are not only rational or logical ways of arguing, but they are also affective. Relatedly, Venäläinen and Calder‐Dawe (2024), have demonstrated how young people's resistance to sexual harassment is infused with affective‐discursive dilemmas of empowerment on one hand and vulnerability on the other. In the Brexit context, Andreouli et al. (2019) found that Remain voters faced an ideological dilemma between supporting and resisting a pro‐EU establishment, while Andreouli and Nicholson (2018) demonstrated that Brexit negotiations among voters involved balancing between safeguarding national sovereignty and valuing international collaboration. Furthermore, Sullivan (2021) identified an affective dilemma among UKIP voters concerning anger about immigration and powerlessness to influence the issue, highlighting the ambivalent nature of the EU.

The concept of affective argumentation as an expansion of Wetherell's (2012) affective‐discursive approach in combination with the principles of rhetorical psychology (Billig, 1987; Billig et al., 1988) allows an examination not merely of the argumentative nature of thought but also affect and emotions in everyday (political) talk. The concept builds and expands on the previous discursive psychological work on emotions as functional (Edwards, 1999) and social (Wetherell, 2012), offering an elaborated and theoretically grounded approach to examine the complex interplay of affect and argumentation in everyday sense‐making around political topics. It allows for grasping not only how discourse and emotions are inseparably intertwined but also to study their dilemmatic and dialogical character in a systematic way. Using the affective‐discursive approach (Wetherell, 2012) and rhetorical psychology (Billig, 1987, 1999) to study the issues related to the EU in Finland, the study aims to first examine *what* the affective‐discursive practices in the populist EU discourse are. More specifically, *how* they are constructed in argumentation and *with what* discursive consequences. Second, it aims to demonstrate the way affect and emotions can be approached, analysed and identified within this political discourse as part of everyday reasoning (Billig, 1987; Wetherell, 2012). Consequently, it provides a nuanced understanding of the affective and dilemmatic aspects of lay political thinking around the EU while contributing to the social psychological study of affect as an interplay of affect, social relations and argumentation.

### Context of the study

The context of this study is Finland, which joined the EU in 1995 following an advisory referendum in which 56.9% of Finnish citizens voted in favour of membership. As a Nordic country, Finland has sought to promote its economic interests within the EU, particularly concerning trade and market access. While most mainstream political parties in Finland generally support European integration, EU scepticism and criticism have been the defining feature of the radical right populist Finns Party (Raunio, 2008, 2011). The party mobilized EU scepticism during the 2011 parliamentary elections amid the EU financial crisis, contributing to its record high electoral success (Herkman, 2017). Previous research has identified three themes in the party's criticism of the EU: the EU as an elitist bureaucracy, national EU policy and integration through increased immigration (Raunio, 2011). Finland's concerns about the EU also relate to the future of agriculture and security (Sakki et al., 2021), with agricultural issues remaining central to its cultural identity (Sakki et al., 2024).

Although the EU has generally been viewed positively by the majority of the population, and trust in the EU has remained relatively high among Finns in the past (European Commission, 2023), opinions and sentiments towards the EU have fluctuated. For example, in 2016, there was a citizens' initiative to hold a referendum on Finland's EU membership. In 2020, the Fixit movement (i.e., Finland leaving the EU) organized a demonstration against the EU in the capital city of Finland, Helsinki, which featured speakers from the FP and Eurosceptic MEPs who demanded Finland's withdrawal from the EU. The interplay of the country's cultural and historical identity, the presence of a strong Eurosceptic party and shifting public opinions make Finland an intriguing context for studying populist lay argumentation.

## METHODS

### Material

The research material comprised 55 semi‐structured interviews conducted in Finland with voters of the populist radical right Finns Party (*n* = 25) and voters of other Finnish political parties (*n* = 30). The high 45% representation of FP voters was related to the project's broader aim of increasing understanding of populist thinking and the appeal of populism. Participants were recruited through their prior participation in a questionnaire study, direct contact and snowballing. They were informed that their participation was voluntary and anonymous. The interviews were recorded and transcribed verbatim. The original Finnish quotes (see Appendix S2) were translated from Finnish to English by the first author, who is a native Finnish speaker and fluent English speaker. The mean duration of the interviews was 1 h 46 min. The study and interview scheme received approval from the ethical committee of the University of Eastern Finland.

The interview comprised 17 main questions related to contemporary affairs and political issues, but the format also allowed the interviewer to ask various follow‐up questions connected with the participant's answers (see Appendix S1 for the relevant interview questions). The study investigated people's thoughts, opinions, attitudes and understandings of the EU and related issues both directly and indirectly. The indirect approach involved the use of images, which function as powerful tools to elicit emotions, negotiate emotional responses and embodied meaning‐making (Byford, 2018; Martikainen & Sakki, 2023). Additionally, we asked participants to comment on a statement from the FP's European parliamentary election campaign. The statement was as follows: ‘The survival of Finnish democracy and the welfare state will not be possible in the coming decades if we cannot break away from the Brussels diktat that affects all areas of life. Europe‐wide bureaucracy does not represent the true virtues of Europeanism’ (Perussuomalaiset, 2019, p. 2). Based on their responses, we divided the participants into three groups: agreeing (*n* = 17), ambivalent (*n* = 14) and disagreeing (*n* = 22). Two participants were excluded as they were not asked this question. To capture the more Eurosceptic and critical voices and illuminate the negotiation of EU issues, the final research material includes all talk related to the EU among individuals who supported or did not oppose the statement (*n* = 31). The majority of respondents were FP voters (*n* = 20), but the participants also included voters from other established parties, such as the Centre Party (*n* = 4), the Christian Democrats (*n* = 3), the Liberal Party (*n* = 1), the Left Alliance (*n* = 1), the Greens (*n* = 1) and one participant who did not disclose their party affiliation. By focusing on these participants instead of FP voters exclusively, we avoided labelling certain party voters ‘populist’, often considered a derogatory category (Rovamo & Sakki, 2024), while gaining access to the broader Eurosceptic populist discourse across the political spectrum.

### Analytical procedure

The identification of affective‐discursive practices, that is, the combination of discursive analysis and affect (Sakki & Pettersson 2016; Tormis et al., 2025; Wetherell, 2012), followed a pragmatic three‐step model of critical discursive psychological analysis (Wetherell, 1998), which includes analysis of content, form and function (Sakki & Pettersson, 2016). It has previously been used to study highly contested and emotionally charged political issues, such as immigration (Sakki & Pettersson, 2016), climate and gender issues in populist discourses (Tormis et al., 2024, 2025), making it suitable for examining affective argumentation that moves beyond the immediate analysis language and connects it with the broader ideological context.

In practice, this model allows us to answer the questions of *what* is talked about, *how* it is talked about and *for what purposes* (Sakki & Pettersson, 2016), aligning with the research questions of how the EU discourse is constructed affectively and with what discursive implications. By following these steps, we performed a detailed analysis of language as action‐oriented and speakers as agentic (Potter, 1998) and were able to examine affect as part of the discourse (Sakki & Martikainen, 2021; Tormis et al., 2024; Wetherell, 2012). We began the analysis of content (not to confuse it with qualitative content analysis; Krippendorff, 2019) by reading and re‐reading the material to familiarize ourselves with it (Potter & Wetherell, 1987), coding the research material to identify different topics. This initial and more descriptive coding revealed how EU talk was related, among other topics, to national independence, the economy, Brexit, bureaucracy and agriculture.

In the analysis of form, we went beyond the descriptive accounts and paid close attention to the discursive forms and rhetorical tools in the participants' accounts, including the use of disclaimers, ‘I'm not against the EU, but…’ (Andreouli & Nicholson, 2018; Potter, 1996), historical narrative (Augoustinos, 2001) and national referents, such as ‘here’, ‘we’, ‘our people’ and so on (Billig, 1995; Condor, 2000), aligning with the rhetorical notion of thinking (Billig, 1987; Billig et al., 1988) as intertwined with feelings and emotions (Billig, 1999). During this phase, we also analysed the discursive construction of affect. In line with Wetherell's (2012) affective practice approach, affect is considered ‘pre‐eminently a relational and social event’ where and the “dialogic” activities involved need to be at the forefront of attempts to understand affective meaning‐making (p. 74). Therefore, we paid attention to subject positions, that is, ‘social construction of particular selves’ and others (Edley, 2001, p. 210) and how they were made relevant through specific—affective and argumentative—ways of talking (Wetherell, 1998). Affect was examined as direct statements of emotions or emotion categories, ‘It makes me angry’ (Edwards, 1999), or implicitly through the use of various rhetorical and discursive tools (Potter, 1996) that enabled the flexible construction of ‘us’ and ‘others’ imbued with related affects. Rather than analysing how contrasting categories of emotion (e.g., dispositional vs. temporary states, honest vs. fake) can be used in interaction to explain human behaviour (Edwards, 1999), the pragmatic three‐step approach allows identifying affect in the dynamic interaction of *social* categories through discursive and rhetorical tools in a specific context.

Finally, in the analysis of function and in line with the critical discursive psychological approach (Wetherell, 1998; Wetherell & Potter, 1988), we analysed the social and political consequences or functions of the affective‐discursive practices (Potter & Wetherell, 1987; Wetherell & Potter, 1988). The identification of discursive functions required us to analyse the affective‐discursive practices in light of their broader social and political context (Edley, 2001; Potter & Wetherell, 1987), transcending mere description and demanding interpretation of the use of rhetorical and discursive tools (Wetherell & Potter, 1988). The difficulty of analysing function arises from the notion that functions are seldom directly available for study and cannot be analysed descriptively or as the speaker ‘sees’ them (Wetherell & Potter, 1988);rather sensitivity is required to the interpersonal, ideological and sociopolitical context.

In sum, the pragmatic three‐step model provided an integrated analytical framework of content, form and functions (Sakki & Pettersson, 2016; Tormis et al., 2025), combining a micro‐level language analysis with the more macro‐level ideological context (Potter & Wetherell, 1987; Wetherell & Potter, 1988).

## RESULTS

Through our analysis, we identified three affective‐discursive practices: (1) bitterness towards the undeserving ‘other’ justifying opposition to the EU, (2) national uncertainty mitigating criticism of the EU and (3) national glory devaluing the EU. Each practice was constructed on various subject positions and affects, and the rhetorical and discursive tools served distinct functions. We illustrate each practice with two examples from the research material that demonstrate the patterned way of talking across the material. Furthermore, in presenting the findings, we retain the colloquial and rhetorical nuances wherever possible. The results are summarized in Table 1.

**TABLE 1:** A summary of affective‐discursive practices in populist EU discourse.

Affective‐discursive practices	Bitterness towards the undeserving ‘other’ justifying opposition to the EU	National uncertainty mitigating criticism of the EU	National glory devaluing the EU
Subject position	Unprivileged, victimized ‘us’; privileged, undeserving ‘other’; the irrational EU	The nation as powerless and ‘in need’, the EU as a powerful entity	A powerful, glorified nation; the EU as an illegitimate political authority
Rhetorical and discursive tools	National referents of identity and place; laughter, economic arguments	Concession, Brexit as a lived memory, economic and sovereignty arguments, facts	National referents of identity and time, master narrative of the nation, positive attributes
Affect	Bitterness, moral anger, national victimhood, unfairness	Discontent, uncertainty and insecurity (outside the EU)	National greatness and nostalgia, glory
Function	Justify and amplify anti‐EU sentiment; criticize and blame the national elite	Mitigate criticism towards the EU; appear rational; justify staying in the EU	Belittle and downplay the value of the EU and its membership; strengthen Finland's sovereignty

### Bitterness towards the undeserving ‘other’ justifying opposition to the EU

The first affective‐discursive practice makes salient a moral divide between the unprivileged ‘us’ and the privileged ‘them’ through the use of national referents (Billig, 1995) and emotion words (Edwards, 1999), which highlights the hierarchical relations between the nation, the EU and Southern Europeans through nationalist talk. In Extract 1, below, the participant—an FP voter—was asked about the current societal situation in Finland. In their response, the participant criticizes the EU and its policies.

**Table:** 

Extract 1	
1	Well, I get angry and frustrated by throwing money abroad that is to the EU, all the
2	support packages, like [I: Mm.] in a way, at least the way in which well we support
3	Southern Europe and then at the same time, the news reports that they're
4	doing a four‐day working week there and so on, and then like here it means
5	an increase in the cost of living and an increase in taxation and like.
6	Perhaps I don't like this direction that, that in a way, we would rather provide for
7	others than (laughs) the people of this country… I don't like this direction like
8	of course we could support and so on if we had everything okay like. [I: Mm] So like, I
9	see that we have kind of given up taking care of our own citizens' life, life
10	conditions like here.

The participant explicitly expresses anger and frustration using emotion categories (1; Edwards, 1999) to construct the EU's irrationality. The individual feelings of anger and frustration are justified through national economic reasoning that echoes similar arguments and the affectivity of righteous anger seen among Brexit Leavers (Andreouli et al., 2019; Sullivan, 2021). Metaphors, such as ‘throwing money’ (1), emphasize the intentionality of these actions, implicitly attributing blame and criticizing national EU politics (Pomerantz, 1978). The participant then juxtaposes two affective subject positions: the moral and deserving ‘us’ and the undeserving, immoral ‘them’ as Southern Europeans (Carr et al., 2019). This is achieved through the use of national referent ‘we’ (2; Billig, 1995), a self‐inclusive strategy of banal nationalism that builds a national identity of morally superior people distinct from the vague group of ‘Southern Europe’ (De Cillia et al., 1999; 2–4). Furthermore, drawing on the news (3) is a way to increase the factuality of the argument and externalize opinion in the form of a collaborative accomplishment (Condor, 2006a). The four‐day working week serves as an often‐used symbol in the research material to illustrate the undeservingness of the ‘other’. In other words, the rhetorical forms employed here function to construct a clear division underscoring the antagonistic and moral dimensions of the intergroup context within this discourse (Carr et al., 2019; De Cillia et al., 1999).

Crucially, this moral juxtaposition reveals a broader normative context where the transgression of equality and meritocracy enables the discursive construction of moral anger, unfairness and bitterness. However, it is not only the undeserving position of the ‘other’ that enables this affect. The participant also rhetorically constructs the victimhood of ordinary Finns through the national referents of place ‘here’ and identity ‘our own citizens’, ‘the people of this country’ (4–9) alongside nationalist claims (6–7). Using the phrasal verb ‘provide for’ (6), along with the distinction between the general ‘others’ and the particular ‘us’, emphasizes the unequal relationship between the two categories, which further functions to justify the anti‐EU position.

Moreover, this example highlights how this positioning is argumentative in nature. The participant draws on the societal norm of helping the disadvantaged using concessions—‘of course we could support and so on’ (7–8; Antaki & Wetherell, 1999)—while simultaneously indicating its conditionality based on the nationalist principle of Finland first (8; Sakki et al., 2024). This allows the participant to appear moral and rational while arguing against supporting the ‘other’. Responsibility for the negative consequences of EU membership is not directly attributed to the EU but to a collective ‘we’ within the nation (7–8). This serves to implicitly attribute blame to the national elite who allows this unfair treatment of ‘us’ (Edwards & Potter, 1992).

Similar to extract 1, extract 2 demonstrates a moral divide, but more directly draws from the victimized position of ‘us’, using embodied laughter to emphasize the affective undertone of bitterness. The extract is a response from a FP voter to the EU‐sceptic statement in the party's European election programme.

**Table:** 

Extract 2	
1	Yeah. Well, here we come to this criticism of the EU so well, of course there it [the EU] has
2	probably brought something good but Finland has paid, paid more there than it
3	has received and we've funded all sorts of. Um, countries like different (laughs)
4	different things where they have moved to a four‐day working week and, and where they
5	have lowered the retirement age and in Finland it's just being raised …
6	What this. Like, like Finland always complies with all of these EU regulations so
7	literally and other countries can do whatever they want and, and in‐ indeed they don't
8	have to follow them and there are no sanctions so we have to be some kind of
9	model country and, like well does it get a feather in your cap but like,
10	it can be said that we Finns have already tortured Finnish people to death (laughs)
11	by these different measures like if I think about like, our agriculture, how, how can
12	they fight against some like Southern European agriculture…

The extract above shows how criticism of the EU is negotiated through the argumentation and counter‐argumentation framework. In practice, the participant uses a concession to acknowledge some of the positive aspects of EU membership, ‘of course …’, which is followed by a ‘but’. This displays awareness of the position's potential flaw and to pre‐empt criticism for overlooking counterarguments (Antaki & Wetherell, 1999; 1–2), thus balancing between criticizing the EU and appearing rational. As in Extract 1, this criticism is based on economic arguments of inequality within the EU (2–4), which serve as a rationalization strategy (Augoustinos, Tuffin, & Rapley, 1999; Wetherell & Potter, 1992) to argue against the Union.

A moral division through national referents (Billig, 1995) again plays a key role in the construction of the victimized position. The participant uses the extreme case formulation ‘always’ (6; Pomerantz, 1986)—to emphasize the moral righteousness of ‘us’ in relation to the privileged ‘other’, transgressing the equality and fairness norm and further reinforcing the construction of bitterness—‘other countries can do whatever they want’ (7). The participant then poses a rhetorical question (9), highlighting the obvious ridiculousness of the situation (Potter, 1996).

Thus, extract 2 demonstrates how national victimhood is constructed using the figure of speech, ‘tortured Finnish people to death’ (10) to depict the harm inflicted upon us. Furthermore, both extracts in this practice feature laughter when constructing the victimized and unfairly treated ingroup (line 7 in Extract 1; lines 3, 10 in Extract 2). This laughter serves both as an embodied affect and a rhetorical form used to soften harsh criticism and emphasize ridicule (Leser & Spissinger, 2020; Sakki & Martikainen, 2021). Regarding content, the extract demonstrates how agriculture became central to portraying the nation's existential battle for survival (11–12). As national agriculture and nature are integral to Finnish identity and serve as a source of pride (Martikainen & Sakki, 2023; Sakki et al., 2024; Tormis et al., 2024) the threat from the external ‘other’, further emphasizes the intergroup antagonism.

To summarize, this affective‐discursive practice demonstrates that the examination of affective argumentation reveals how affect is identified within hierarchical social categories and boundary work between the victimized nation and the privileged ‘other’, constructing them mutually excluding and antagonistic. Nationalist and economic arguments, when rhetorically balanced with concessions, highlight moral transgressions against equality and fairness norms, enabling the construction of unfairness and bitterness towards ‘them’. This way of constructing affect and argumentations functioned to justify and amplify the anti‐EU position, as well as criticize the national political elite.

### National uncertainty mitigating criticism of the EU

Contrary to the previous affective‐discursive practice that was built on antagonistic relations, this practice demonstrates how the EU and Finland are portrayed as interdependent through managing an affective dilemma (Venäläinen & Calder‐Dawe, 2024; Wetherell et al., 2020) of discontent with the present in the EU and uncertainty about leaving the EU. Extract 3 is a response to the FP statement by a Christian Democrat voter.

**Table:** 

Extract 3	
1	Well I agree about the bureaucracy, that it's um, really heavy machinery. Um like, still
2	the principle of local decision‐making should, like the principle of local decision‐making
3	should be implemented better, um so some things, I'm not maybe like completely.
4	that we have to let go completely, but like is there any other option than to completely
5	or all or nothing. But like maybe I would wait and see what Brexit looks like
6	like after a few years, and then maybe draw new conclusions. But like at this point, I
7	wouldn't dare to. Dare to like suggest anything more radical.
8	But like that I agree that it's heavy and difficult machinery and because it's like,
9	because our, hands are completely off of it,
10	like because we've handed over the reins so do we notice it like ourselves
11	So like that's maybe, maybe a bit like scary.

The participant explicitly agrees with the FP statement by portraying the EU as bureaucratic (1), which is a common criticism of the bloc in Finland (Sakki, 2010). The rhetorical use of the modal auxiliar verb ‘should’ emphasizes the national decision‐making processes currently hindered by the EU (2–3), portraying it as a distant but powerful political authority. However, the participant uses a disclaimer (Hewitt & Stokes, 1975) to mitigate the criticism of the EU by questioning its dichotomous nature—‘I'm not completely, like that we had to let go … is there any option … or all or nothing’ (4–5), which serves to cause the speaker to appear more balanced and rational on the issue.

The negotiation between criticism and a lack of alternatives reveals an affective ideological dilemma (Billig et al., 1988) between discontent and uncertainty in which the EU is depicted simultaneously as failing to serve the nation and its people, while withdrawal is presented as risky and irresponsible. Rhetorically, the dilemma is managed through repetitive use of disclaimers ‘but’ (4, 5, 6, 8), which allows the speaker to anticipate and manage counterarguments. From the perspective of affect, this engagement of (counter)arguments enables the construction of uncertainty and worry through the *potentiality* of negative consequences outside the EU. Furthermore, portraying leaving the EU as ‘radical’ (7) resembles the rhetoric of the Remain supporters in the Brexit debates (Andreouli & Nicholson, 2018), which further serves to soften criticism of the EU.

Moreover, the extract illustrates a common pattern in which Brexit is used as a living memory (Condor, 2006b; Liu & Hilton, 2005), which further problematizes leaving the EU and emphasizes the affect of uncertainty: ‘maybe I would wait and see what Brexit looks like’ (5–7). The use of Brexit as a discursive tool and minimizing words, including ‘maybe’, not only softens the criticism but also functions to justify staying in the EU. However, the participant then returns to the FP's statement and explains criticism of the EU by positioning Finland as powerless through sovereignty arguments ‘because our hands are completely off of it’ (8–10), reflecting again the Brexit debates, in which the UK's powerlessness was used to criticize the EU's authority (Andreouli et al., 2019; Sullivan, 2021). The participant then poses a rhetorical question ‘so do we notice it like ourselves’ (11; Potter, 1996), inviting critical reflection on the loss of national power within the EU (Sullivan, 2021). An explicit emotion word ‘scary’ (12) is further used to strengthen this present discontent and loss of sovereignty as an EU member. Analytically, the subject positions of the EU as a distant, yet powerful political union and of the powerless nation are constructed through the argumentative framework of Brexit, sovereignty claims and disclaimers. Together, they reinforce the affect of uncertainty and worry, which serve to argue in favour of remaining in the EU.

While extract 3 indicated how the speaker balanced between criticizing the Union while arguing for staying in the EU, extract 4 emphasizes the interdependent relation between the nation and the EU. It draws similarly on Brexit, but uses facts and economic arguments to justify the position of a nation in need. The extract is derived from a discussion with a Centre Party voter about the future of Europe and the world.

**Table:** 

Extract 4	
1	So in the EU it may happen that like there will be other these other like add‐ will
2	be F‐ Fi‐ Fixit then and, so we will start like to separate from that EU and well like.
3	Alone we wouldn't like that would be again economically bad if we weren't in the
4	EU so like these internal markets and others it's a double‐edged sword like
5	today I read, I just read from yesterday's Hesari [the largest subscription newspaper in
6	Finland] that like in England it's difficult even to get petrol because like the foreign
7	drivers won't drive there, and they don't have a car, like tanker drivers themselves, so
8	they have started to have these problems now.

The extract shows how national insecurity is rhetorically embedded in the probabilistic conditional clause ‘it may happen’ (1), which depicts the future as uncertain (Cap, 2021). The use of the Finnish equivalent of Brexit, ‘Fixit’ (1–2), works as an anchor and a concrete example to describe the movement against the EU. The subject position of a nation powerless and potentially ‘in‐need’ is achieved through economic arguments: ‘Alone … would be again economically bad if we weren't in the EU’ (3). As in extract 3, the participant negotiates EU issues by using the expression ‘a double‐edged sword’ (4), positioning the participant as critical yet balanced by acknowledging both negative and positive aspects of the EU membership.

From the perspective of affective argumentation, the dilemmatic negotiation of power serves to construct dependency between the nation and the EU, further justifying remaining in the bloc. This dilemmatic negotiation also enables the participant to distance themselves from the extreme or radical views of ‘Fixit’ by drawing on Brexit as a negative example of problems outside the EU (6–8). Similar to extract 1, the participant draws on news, but with a different goal: to bolster factuality and strengthen the claim about the potential threats of leaving (Condor, 2006a).

To summarize, this affective‐discursive practice demonstrates how affective argumentation is built through an interdependent relation: between the powerless nation and the powerful EU. This interdependency highlights the negotiation of an affective dilemma between criticizing the EU (as an economic power) and justifying remaining in the EU. The use of economic and sovereignty arguments, facts, concessions and Brexit as living memory ties argumentation to the affects of national uncertainty, insecurity and worry. Therefore, examining affective argumentation illuminates how affect and identity work allow mitigating criticism of the EU.

### National glory devaluing the EU

Unlike the previous practice that portrayed the nation ‘in‐need’, the third affective‐discursive practice focuses on the nation and its historical continuity as a strong country (Obradović & Bowe, 2021), independent from the EU. It draws on the typical rhetoric of nationalist movements, constructing an idealized image of the past which is juxtaposed with the degraded present, powering and legitimizing a nationalist future (Levinger & Lytle, 2001; Hakoköngäs et al., 2020). Extract 5 is derived from a response to the FP statement from a FP voter.

**Table:** 

Extract 5	
1	And after all, we were doing well, except now a war was fought in between, by
2	negotiating, making deals, and living but does it bring this EU or some,
3	like, this big alliance, does it really bring anything [I: Mm]
4	Like if also this Finland, so then like this is a wise country after all. So, we've managed
5	for a hell of a long time without this, this of course we got the paymaster's role
6	and, we got some pretty damn good jobs there [I: Mm]…
10	so it's up to each country to decide how to do. And likewise their laws and others.
11	Decide for themselves without anyone pressuring.

The participant first constructs a strong nation (1–2), followed by a rhetorical question – ‘but does it … does it really bring anything’ (2–3; Potter, 1996), which is used to raise a point and question the relevance of the EU. Furthermore, past challenges and struggles, such as war, alongside positive attributes—‘this is a wise country after all.’ (4)—work as merits to construct a master narrative of national success (Hakoköngäs et al., 2020; Sakki et al., 2024). The use of national history as a rhetorical device (Kirkwood, 2019; 4–5) and national referent of place ‘this Finland … this … country’ (4) and identity ‘we’ (4–5; Billig, 1995) construct the subject position of a strong and powerful nation (Augoustinos, 2001; Liu & Hilton, 2005; Pettersson & Sakki, 2017; Reicher & Hopkins, 2001). This subject position further enables the discursive construction of national pride, which serves to devalue the EU and its membership.

The participant uses irony—‘of course we got the paymaster's role’—and concessions (5–6; Antaki & Wetherell, 1999), which demonstrates how the affective argumentation framework is built through the continuous argument‐counterargument setting, which functions to criticize the EU as an economically unfavourable deal for Finland. Furthermore, the participant draws on nationalist arguments (10–11) without explicitly criticizing or blaming the EU. Nonetheless, they implicitly portray the EU as an external political authority exercising unwarranted power.

Extract 6 is from a FP voter's response to the FP statement. While the participant draws on similar arguments of sovereignty and temporal comparison to those in extract 5, they position the EU more clearly as an illegitimate and irrational authority:

**Table:** 

Extract 6	
1	Yeah because there [in the EU] these things are determined that like here may not be
2	maybe like fulfilled, and these regulations and so on, so it's it's madness indeed
3	we've managed here before so we should manage now too so they can't dictate,
4	dictate how we go about things, especially in Finland as this has always been
5	independent and like this so … from there [the EU] all of this non‐nonsense,
6	gibberish is coming that hasn't been before.

The participant depicts the EU as an authority detached from the national reality (1–2). Again, national referent of place (Billig, 1995) ‘here’ and ‘there’ (1) are used to distinguish the nation and the EU as distinct independent categories, distant from each other. Vague expressions, such as ‘these things’ (1) and ‘so on’ (2), serve to distance the speaker from the practicalities and details of EU issues (Augoustinos, 2001). This example demonstrates the affective construction of national greatness through temporal comparison in which the present is constructed against the glorified past (Condor, 2006b; Sullivan, 2021)—‘we've managed here *before*’ (3). This provides a further justification for the future prediction (3; Palmer, 2001) and functions to depict a strong nation (in case of leaving the EU). Besides portraying the EU as a reality‐detached authority, negative expressions, such as ‘dictating’ (3–4), construct it as a strong top‐down entity, reinforcing the argument against it.

The use of national history and temporal markers of national identity (4; Pettersson & Sakki, 2017; Sakki et al., 2024) construct the continuation of national identity and the master narrative (Obradović & Howarth, 2018; Sakki et al., 2024), which enables the construction of national pride and glory. Emotive expressions of extreme case formulation (Pomerantz, 1986): ‘it's madness indeed’ (2) and drawing on the EU's irrationality (5–6), are used to build rationality for the speaker and devalue the EU as a political authority.

In sum, this affective‐discursive practice demonstrates how affective argumentation operates by distinguishing the glorified nation and the EU as independent social categories. This category distinction is achieved rhetorically through using national referents of identity and time, positive attributes and a master narrative of the nation. Portraying the categories as distinct and distant enables the affective constructions of national greatness and nostalgia, which work not only to devalue the EU but also to potentially counter future uncertainties outside the Union.

## DISCUSSION

This study examined the affective‐discursive practices of populist EU discourse among Eurosceptic voices in Finland, highlighting their social and political implications. We aimed to address a gap in social psychological research by placing affect and emotion at the forefront of the discursive and rhetorical psychological analysis of the EU issues. Consequently, we sought to demonstrate how argumentation is bound up with subtle affects and can be analysed as part of everyday reasoning in political discourse.

With respect to the first research aim, we identified three affective‐discursive practices: (1) bitterness towards the undeserving ‘other’ justifying opposition to the EU, (2) national uncertainty mitigating criticism of the EU and (3) national glory devaluing the EU. The first practice constructed a moral divide and hierarchical relation between a victimized but morally superior ‘us’ and an undeserving ‘them’ (as Southern Europeans) and the political elite. The examination of affective argumentation showed how national referents of identity and place (Billig, 1995), economic arguments (Augoustinos, Tuffin, & Sale, 1999) and laughter as an embodied form of affect (Wetherell, 2012) were used in the construction of these subject positions. This finding echoes a logic akin to that of the radical right populist discourse of otherness and anti‐elitism (Sakki & Pettersson, 2016) and the arguments of ‘national interest’ used in Brexit referendum debates (Hunt & Demasi, 2025). The nationalist arguments, rooted in the principle of meritocracy and equality, served to construct the affects of unfairness, bitterness and moral anger, justifying and amplifying anti‐EU sentiments. Furthermore, this practice was strongly built on the ‘sacred’ Finnish agriculture threatened by the external ‘other’ and the political elite (Sakki et al., 2024; Tormis et al., 2024), which further reinforced the construction of moral anger.

The second practice depicted an interdependent relationship between a powerless nation and a powerful EU as a political entity. This practice demonstrated how rhetorical tools, such as disclaimers (Hewitt & Stokes, 1975), concessions (Antaki & Wetherell, 1999) and minimizations, together with the discursive use of Brexit as a living memory, strengthened the *potentiality* of the negative consequences of withdrawal and thereby functioned to construct discontent towards the present within the EU and uncertainty about the future outside it. Consequently, this affective argumentative framework served to justify remaining in the EU and soften the most extreme opposition to it. The findings align with prior research on the EU and Europe as ambivalent and contested categories that are used to balance between economic pragmatism and political interference in a national context (Andreouli et al., 2017; Andreouli & Nicholson, 2018; Sullivan, 2021).

The third practice positioned the nation as strong and powerful and the EU as an illegitimate authority, building a clear distinction between the two categories. Examining affective argumentation within this practice revealed how this national identity was built rhetorically through national referents of time and identity (Billig, 1995) and national master narratives of historical success (Hakoköngäs et al., 2020; Pettersson & Sakki, 2017; Sakki et al., 2024), which served in the construction of national greatness, pride and glory. It is worth noting that despite the different context of Sullivan's (2021) study in Britain with UKIP voters, the first and third affective‐discursive practice in this study reflected remarkably their way of arguing. Such arguing highlighted the positive experiences from the past when things were (economically) better and the decline that followed accession to the EU. Furthermore, the moral national superiority in the first practice and the historical glorification in the third practice resemble the psychological notion of the anti‐social triad of grievance politics (Capelos et al., 2025; De Zavala et al., 2009), including collective narcissism, which might explain the hostility and negative intergroup bias towards the ‘other’ (De Zavala et al., 2009). However, the present study analysed EU talk as part of the broader societal structures and context, indicating that the idealization of the nation through a master narrative (Sakki et al., 2024) was used to strengthen the nation's position, which subsequently allowed devaluing the EU as a political authority and mitigating the potential consequences of leaving the Union.

In summary, the subject positions of the nation as (1) victimized and unprivileged, (2) powerless and in need or (3) strong and sovereign—shaped through various rhetorical and discursive tools—play an essential role in constructing affect. This affect can either amplify criticism and anti‐EU sentiment or justify remaining in the EU and maintain the status quo. These findings align with previous research demonstrating how temporality, history and national referents, are key rhetorical tools for defining who ‘we’ are as a nation (Augoustinos, 2001; Billig, 1995; Condor, 2006b; De Saint‐Laurent & Obradović, 2019; Kirkwood, 2019; Sakki et al., 2024) and how the nation serves as an affective community through which understanding of the EU is constructed (Sakki et al., 2024).

With respect to the second research aim, previous research on affective practices has tended to focus explicitly on affective phenomena or on certain relevant affects (Haugestad & Carlquist, 2025; Van Der Merwe & Wetherell, 2020; Venäläinen, 2022; Venäläinen & Calder‐Dawe, 2024; Wetherell et al., 2020). By expanding Wetherell's (2012) affective practice theory through the notion of affective argumentation, we demonstrated how affect is enacted in everyday political argumentation, underscoring its usefulness in examining contested political issues, such as the EU. We argue for the dual nature of affect in which on one hand, emotions and feelings can be explicitly employed as rhetorical tools in argumentation to accomplish social acts or functions (Edwards, 1999; e.g., Extracts 1 and 3) and on the other hand, rhetorical and discursive tools can be used to construct, contrast and distinguish identities and intergroup relations that are imbued with contextual notions of affects and tied with broader political and ideological functions (e.g., Extracts 2, 4, 5, 6).

Thus, unlike the straightforward analysis of emotion categories and words in discourse (Edwards, 1999), affective argumentation considers the dialogical and at times dilemmatic character of emotional discourse. It offers an analytic lens to study such emotion talk systematically, placing both affect and argumentation centre‐stage. It further aligns with Cromby's (2019) notion that *all* thinking is *felt* thinking, as feelings constantly shape thinking and vice versa. From this perspective then, the use of economic arguments and fact constructions is not considered ‘rational’ or non‐emotional acts, but as affective practices that allows the construction of bitterness towards undeserving ‘other’ or uncertainty for leaving the EU, irrespective of their veracity. It enables examining affect beyond the dichotomic conceptions of positive/negative, constructive/destructive, rational/irrational (Leser & Spissinger, 2020), highlighting how they are embedded in nuanced constructions of identities and relations.

Furthermore, while previous quantitative studies have identified emotions, such as resentment, victimhood and nostalgia, key in reactionary politics which adopts a bitter outlook to political life and is expressed through oppositions to the EU (Capelos et al., 2025; Capelos & Katsanidou, 2018), this study adds *qualitative* and bottom‐up understanding about the *content* of such psychological constructs and emotion categories through analysing the argumentation of laypeople. This study indicated how these relevant affects, particularly bitterness, unfairness, victimhood and nostalgia, were constructed contextually and socially between the nation, the EU and other countries. Therefore, it aligns with other qualitative discursive studies that have analysed the emotionally laden Brexit discussions (Andreouli et al., 2019; Sullivan, 2021) but also indicates the contextual nuances in a Nordic context. Future social psychological research could apply an affective‐discursive and affective argumentation approach to other timely and contested political issues, using different qualitative materials. For example, ‘go along’ interviews in public (Wetherell et al., 2015), conversations at home (Billig, 1991), or political campaigns could yield a richer understanding of the interplay between multiple societal actors, including the politicians, the media and the audience.

This study is not without limitations. The data was gathered in 2021, meaning that the most recent political events, which could have impacted the content of populist EU discourse, were not included. However, our focus was not the content of the discourse per se but language use and argumentative constructions of affect, using the EU as the context. We therefore believe that our study represents a valuable contribution to the field of discursive social psychology by demonstrating in detail how political argumentation and affect are bound with sociopolitical context with specific functions. A further limitation is that a single country context may limit the generalizability of the results. Nonetheless, the findings may prove relevant in similar socio‐demographic and political settings, such as Sweden, encouraging cross‐country comparative research initiatives.

Lastly, recent EU elections (in 2024) indicated a rise in Eurosceptic far‐right parties. Yet, our findings in the Finnish context suggest that, despite the constructions of bitterness, moral anger and unfairness towards the undeserving ‘other’ in the populist EU discourse, there was limited outright opposition to the EU. Instead, the examination of affective argumentation illuminated the hierarchical, interdependent and independent social relations between the nation and the EU. This further highlighted a dilemmatic negotiation between present discontent and future insecurities, with national pride and greatness serving as a potential buffer and protection against these insecurities. Therefore, bringing both affect and argumentation to the forefront of political lay discourse can enrich the traditional discursive psychological analysis of emotions, deepening our understanding of such discourses and their potential social and political consequences.

## AUTHOR CONTRIBUTIONS

**Helenor Tormis:** Conceptualization; writing – original draft; methodology; writing – review and editing; formal analysis; visualization; validation; project administration. **Inari Sakki:** Conceptualization; funding acquisition; writing – review and editing; supervision; methodology. **Katarina Pettersson:** Writing – review and editing; supervision; conceptualization; methodology.

## FUNDING INFORMATION

This research was funded by the Kone Foundation (Grant No. 201906612).

## CONFLICT OF INTEREST STATEMENT

The authors have no conflicts of interest to declare.

## Supporting information


Data S1.

## Data Availability

The data that support the findings will be available at the Finnish Social Science Data Archive for research and educational purposes after the project is finished.

## Footnotes

^1^
Wetherell (2012) distinguishes between ‘emotion’ as a conventional cultural category and affect as a more embodied construct embedded in everyday discursive practices and interactions. While acknowledging this, the present study aligns with previous research (e.g., Sakki & Martikainen, 2021; Wetherell et al., 2020) and uses ‘affect’ and ‘emotions’ interchangeably to emphasize their relational and contextual nature while drawing on the (social)psychological literature on emotions.
