# Supplementary material for: National bitterness, powerlessness and greatness: Examining constructions of affect as part of argumentation in populist EU discourse in Finland
Source: Br J Soc Psychol. 2026 Feb 12;65(2):e70055. doi: 10.1111/bjso.70055 (PMC12900993; doi:10.1111/bjso.70055)
Supplement: Supplementary file 1 — Data S1. [file BJSO-65-0-s001.docx]

**Appendices**

Appedix 1. The Original Extracts in Finnish

| Extract 1, a FP voter | No, suututtaa ja turhauttaa rahan syytäminen sinne ulkomaille eli EU:hun kaikki tukipaketit niinku … tavallaan ainaki se tapa niinku millä, millä tota, me tuetaan Etelä-Eurooppaa ja sitte samaan aikaan, uutisoidaan et siellä tehään nelipäivästä työviikkoo ja, ja muuta ja sitte tota meillä nää tarkottaa elinkustannusten nousua ja, ja verotuksen nousua ja niinku tavallaan. En ehkä tykkää tästä suunnasta että, että tavallaan, elätetään mieluummin muita kun (naurahtaa) tämän maan, maan ihmisiä … Tästä suunnasta en pidä et toki voiaan tukee ja muuta jos itsellä olisi kaikki niinku kunnossa että. Et tota, mä nään että me ollaan vähä niinku luovuttu siitä omien kansalaisten. Elämän, elämän edellytysten. Hoitamisesta niinku täällä. |
| --- | --- |
| Extract 2, a FP voter | Niin. No, tässä päästään tähän EU-kriittisyyteen niin tota, toki sielt on varmaan niinku jotain hyvääki tullu mut kylhän niinku Suomi on, enemmä maksanu, maksanu sinne kun mitä on saanu ja ollaan rahotettu kaiken maailman. Ööh, maiden tota erilaisia, (naurahtaa) erilaisia asioita missä on siirrytty nelipäiväseen työviikkoon ja, ja mis on madallettu tota eläkeikää ja Suomessa vaa korotetaa ... Mitä sil niinku. Et, et Suomi noudattaa näitä aina näitä kaikkia EU:n säädöksiä niin, kirjaimellisesti ja muut maat voi sitte tehä mitä haluaa ja, ja tosi-, tosiaa hein ei tarvii niit noudattaa ja sielt ei sit kuitenkaa tuu mitää sanktiota et mein pitää olla joku semmone ihmeellinen mallimaa ja, ja tota et saaks siitä jonku sulan hattuun sitte mutta tota, et saa sanoa et ollaan suomalaisia nyt ollaan niinku, kidutettu suomalaiset jo hengiltä (laughing) näillä erilaisilla toimilla et jos mä mietin niinku, meiän maataloutta miten, miten ne pystyy taistelemaan tai että jotai eteläeurooppalaista maataloutta vastaan… |
| Extract 3, a Christian Democrat voter | No tosta byrokratiasta mäkin oon kyllä samaa mieltä, että se on siis, öö todella.Raskas koneisto. Öö että, et, et niinku edelleenkin sitä et se lähi-, lähipäätösperiaate, pitäis niinku, lähipäätösperiaate pitäis niinku toteutua paremmin, öö et jotkut asiat, en mä ehkä niinku ihan kokonaan. Sitä että täytys niinku kokonaan päästä irti, mutta että. Onko siinä sitte muuta vaihtoehtoo kun niinkun kokonaan, tai, kaikki tai ei mitään,mut et ehkä niinkun mä odottelisin että mitä toi. Mitä toi niinku Brexit, tällain niinku muutaman vuoden, jälkeen näyttää. Ja ja sit ehkä tekis uudet johtopäätökset. Mutta että en niinku täs, täs vaihees ehkä kyllä uskaltaisi lähteä. Lähtee niinku ehdottelee mitään radikaalimpia. Mut että siit-, siit-, siitä oon niinku siis sillee samaa mieltä että se on. Raskas ja vaikee koneisto ja koska se on sitten niinku, koska meijän, kädet on sieltä täysin irti, niinku että koska me ollaan annettu langat pois niin, niin huomataanko me sitä niinku itekään, että se on ehkä. Ehkä sillain vähän niinkun, pelottavaa. |
| Extract 4, a Center Party voter | Nii EU:ssa saattaa tapahtua sitten että tota tullee niitä muita, niinku lisä-, tulee, F-Fin- Finexit sit ja, et me aletaan niinku erota siitä EU:sta ja, ja tota noin niin toi. Yksin me ei kuitenkaan niinkun sit se on taas taloudellisesti huono jos ei me olla EU:ssa et siis näitten sisämarkkinoitten ja muitten kannalta et se on kakspiippunen juttu et tänäähä mä luin, luin just eilisest Hesarista että tota Englannissa nyt ei tahdo saada bensaakaan kun tota noin niin ulkolaiset kuljettajat ei aja sinne ja, ja tota heil ei oo auton, re- siis säiliöauton kuljettajia itsellä et tota noin niin heillä alko nää ongelmat nyt sit. |
| Extract 5, a FP voter | Ja, kuiteskin me, tulimme toimeen, paitsi nyt joku sota otettiin välillä neuvottelemalla, kaupoista sopimalla, ja elämällä. Mutta tuopiko se sitten tämä, EU tai joku, niinkun, tämmönen iso liitto. |
| Extract 6, a FP voter | Niin koska sieltähän [EU:sta] määritellään semmosia asioita mitkä niinkun täällä ei ehkä ehkä, niinkun pysty toteuttaan, ja niitä määräyksiä sun muuta niin son se, se on hulluutta kyllä‬ täällä on pärjätty ennenkin niin pitäs pärjätä nyttenkin että eihän ne voi siellä sanella, sanella miten tuota, missäkin mennään, varsinkaa Suomessa ku tää ollu aina itsenäinenja tämmönen niin … Sieltä näillä sieltähän ne tulee kaikki nämä, hö-höpötykset höpinät mitä ennen ei oo ollu. |

Appedix 2. Interview questions that were relevant for the EU talk (translated by the first author from Finnish to English)

Questions written in bold font were the main questions that were asked in all interviews. The questions written in body text were follow-up questions planned to elaborate on the main questions.

1. **What do you think about your current life situation?** What things make you happy or unhappy, what do you enjoy, and what challenges do you face in your life?

2. **How do you see the current situation in Finland?**

When you think about the current situation in Finland, what things make you happy? Can you give an example? What things make you angry or frustrated? Can you give an example? When you think about the current situation in Finland, do you think things are going well or badly? Why/why not, what could be better? What reasons might have led to this situation?

3. **How do you see Finland's future?** Why do you see the future that way?

Next, I would like you to broaden your perspective. **How do you see the future of Europe or the world?** What about your own future? How do you think it will turn out?

4. **Would you like to tell me which party you voted for in the last parliamentary/municipal elections? Did you vote primarily for a party or an individual candidate?**

Could you tell me how you ended up voting for that party/candidate? What appeals to you about that party? Have you voted for another party in the past? If so, which one? Why did you decide to change parties? What is the difference between the right and the left? How actively do you follow politics? How do you typically follow politics? How actively do you participate in politics? How do you participate? What are your opinions of the established political parties?

5. **I will now read you a few sentences taken from the municipal election program (2021) and the European election program (2019) of the Finns Party. I would like you to comment on these statements.**

a. Suvivirsi and Christmas celebrations are part of Finnish culture and schools.

b. The current overheated debate on climate change forms a threat to Finnish welfare.

c. Asylum immigration is both harmful to the receiving society and an ineffective way to help.

d. Municipalities should not get involved in the fuss of small but loud ideological groups. We do not support wasting money on gender-neutral road signs, continuous campaigns against discrimination and racism, or artificial emphasizing of gender issues. We should not compromise on Finnish culture because of overly offended people.

e. The survival of Finnish democracy and the welfare state will not be possible in the coming decades if we cannot break away from the Brussels diktat that affects all areas of life. Europe-wide bureaucracy does not represent the true virtues of Europeanism.
